# The role of intergenerational family stories in mental health and wellbeing

**DOI:** 10.3389/fpsyg.2022.927795

**Published:** 2022-09-27

**Authors:** Alexa Elias, Adam D. Brown

**Affiliations:** ^1^King’s College London, Institute of Psychology, Psychiatry and Neuroscience, London, United Kingdom; ^2^London School of Hygiene and Tropical Medicine, London, United Kingdom; ^3^Department of Psychology, New School for Social Research, New York, NY, United States; ^4^Department of Psychiatry, New York University School of Medicine, New York, NY, United States

**Keywords:** memory, intergenerational, mental health, family, wellbeing

## Abstract

Patterns of memory sharing begin early in one’s life, informing relationships, one’s history, and one’s sense of cultural belonging. Memory sharing among families has been the focus of research investigating the relationship between mental health and intergenerational memory. A burgeoning body of research is showing that intergenerational knowledge of one’s family history is associated with positive mental health and wellbeing. However, research on the specific mechanisms and potential applications of such findings are just beginning to emerge. In particular, studies examining intergenerational storytelling point to the importance of culture and gender as critical factors underlying how stories are told and the extent to which these stories are associated with wellbeing. Such findings hold important promise for the pentation and treatment of mental health issues. As research in this area continues to evolve, the identification and characterization of factors and mechanisms underlying intergenerational family stories and wellbeing may help to guide the integration of family stories into mental health interventions.

## Introduction

Life stories are central to one’s sense of self and the social world that they inhabit. Indeed, considerable research indicates that storytelling is fundamental to human development, cultural frameworks, and the cultivation of relationships (Bruner, 1990; Fivush, 2008). Perhaps this is due, in part, to patterns of memory sharing that begin early in one’s life which inform relationships, one’s history, and one’s sense of cultural belonging (or identities; e.g., McAdams, 2001; Nelson and Fivush, 2004; Fivush and Nelson, 2006). Although historically, within the field of psychology, considerable emphasis was placed on how individuals recall memories decontextualized from social interactions (Hirst and Manier, 2008), there now exists a substantial body of work documenting the cognitive characteristics and outcomes associated with socially-shared memories. Within the social dynamics of a conversation, a narrative recounts the unfolding of an event beyond its literal description. It provides the listener with a contextual understanding of when an event took place, what it was about, and how it may have psychologically and materially impacted those involved (Fivush, 2008). Moreover, for the speaker, recalling one’s own memory to another person may lead to the consolidation and adaptation of the original memory based on a number of factors associated with the speaker and listener (e.g., Hirst and Echterhoff, 2012). Over the course of a conversation, both speakers and listeners are engaged in processes of interpretation that take place during the act of narrative sharing, in which one evaluates the event and the extent to which it may be personally relevant or informative (Conway et al., 2004; Fivush, 2008). These processes not only contribute to the distribution of information between individuals, but subsequently shape a range of cognitive, affective, and decision-making processes (Hirst et al., 2018). For example, there now exists robust data showing the impact of social remembering across a wide range of contexts including eyewitness identification (e.g., Wright et al., 2000; Gabbert et al., 2003), international sales (e.g., Yuan et al., 2010), air traffic controllers (e.g., Smith-Jentsch et al., 2009), emergency responses (e.g., Majchrzak et al., 2007), and medical decision making (e.g., Coman et al., 2013). Taken together, these findings have shown that memory processes across diverse settings are impacted by complex social dynamics which may facilitate recall, forgetting, and subsequent behaviors. Social memory practices also appear to play a role in emotional processes and mental health. For instance, Brown et al. (2012) found that symptoms of forgetting associated with posttraumatic stress disorder (PTSD) may be linked with alterations in how individuals with PTSD process information during acts of social remembering. In particular, in two socially-shared retrieval induced forgetting (SS-RIF) tasks, combat veterans with PTSD exhibited greater forgetting compared to those without PTSD and non-trauma exposed civilian participants.

## Intergenerational memory and mental health

One context in particular in which important social memory sharing may take place is among families. Family narratives have the potential to enrich and contextualize one’s knowledge and understanding of how past generations may have had an impact on one’s current life (Fivush, 2008). In fact, family stories are central to how families interact with one another (Bohanek et al., 2009). Family storytelling has been shown to play an important role in the development of self-identity and one’s participation in the transmission and construction of their family identity (Nelson and Fivush, 2004; Fivush, 2013). However, the purpose, or function, of intergenerational family stories goes beyond the mere sharing of details. Like individual memory (Bluck et al., 2005), there are often explicit and implicit goals associated with the intergenerational transmission of family stories. For instance, older adults may share stories with younger generations to teach lessons and offer cautionary tales (Ryan et al., 2004) whereas younger individuals often engage in social memory sharing with caregivers to make meaning and connect their own experiences to a broader context (Pratt et al., 2008). Additionally, intergenerational family stories may be a catalyst for increasing cohesion among family members, especially as children age and begin to take active roles in contributing to the intergenerational recounting (Norris et al., 2004).

In fact, there is now a growing body of evidence indicating that the process of intergenerational transmission of family stories is critical to not just one’s identity, but to their mental health (Duke et al., 2008; Fivush et al., 2011; Svob and Brown, 2012; Chen et al., 2021). For instance, past studies have found that young adults who can recall their parents’ history of living through conflict, such as violent political upheaval, experience a personal, positive life-changing effect related to their perceived importance and transitional impact when compared to young adults who can recall non-conflict related family history (Svob and Brown, 2012). Additionally, intergenerational biographical knowledge has been associated with psychological wellbeing as measured on self-report measures (Duke et al., 2008; Fivush et al., 2011). Studies on family narrative sharing and young adults provide evidence to show that adolescents who know more about their family history tend to have less anxiety, higher self-esteem, more locus of control, better family functioning, and less behavioral problems (Duke et al., 2008). Furthermore, adolescents whose families share more narratives tend to have higher emotional wellbeing overall (Fivush et al., 2011). Specifically, in a study of parent and child dinner conversations, greater engagement among mothers during conversations was associated with lower internalizing behaviors among the children (Fivush et al., 2011). Relatedly, Zaman and Fivush (2011) studied family narratives for perspective-taking (e.g., use of cognition and affect words) in relation to wellbeing. In this study, girls who recalled memories from the perspective of their mothers exhibited fewer internalizing and externalizing behaviors on the Child Behavior Checklist. More recently, Adler et al. (2016) found coherent turning-point events and those who resolved negative life events to be associated with reported lower depression and higher self-esteem and life satisfaction.

Additionally, research on the mechanisms underlying the role of intergenerational family memories and wellbeing would benefit from further research, given initial findings showing that for example, attachment style as well as the structure and content of memories may play an important role in this process. Zaman and Fivush (2013) found that the coherence and affective process expressed in intergenerational stories were significantly correlated with adolescents’ secure attachment. Along those lines, intergenerational stories that are more coherent and generated by adolescents about their parents have been found to be associated with higher wellbeing scores (Merrill and Fivush, 2016).

The link between family background knowledge and psychological wellbeing, particularly amongst younger generations, sheds light on the critical role of intergenerational family narrative sharing on psychological wellbeing (Duke et al., 2008; Fivush et al., 2011). Importantly, recent work suggests that the potential mental health benefits of intergenerational narratives can be found outside of heteronormative and biologically-based concepts of family. For example, in some circumstances, individuals who may be marginalized or rejected by family members may have limited access to stories from their biological family and instead benefit from stories deriving from “chosen families.” Preliminary work aimed at understanding social memory practices in more diverse family constructs with queer-identifying and heterosexual women demonstrated that family knowledge was only associated with lower levels of depression in heterosexual women, whereas knowledge about a chosen family member (e.g., one’s “closest friend”) had that same effect on both heterosexual women and sexual minority women (Gardella et al., 2021).

### Emergence of intergenerational memory

One particularly fascinating aspect of family narrative research investigates the way in which one acquires biographical knowledge. It is common for one to know at least some details about their family history, such as where their ancestors originated from, or how their parents met; however, it is easy to take this knowledge for granted, as one might not consider how knowledge of such family stories may have an impact on the way we carry out our day to day lives. Family knowledge may be acquired throughout one’s life, beginning in childhood, through processes of intergenerational narrative transmission in which family stories are told from generation to generation. These stories are often recounted in many parts of our lives, sometimes during our most mundane and routine interactions that take place at dinner conversations, in transit, and while running errands (Fivush, 2008).

A growing body of research investigating the development and transmission of family stories indicates that parent–child communication strategies and the transfer of biographical knowledge is situated and influenced by gender. This may impact the way individuals share and interpret their own autobiographies. Although gender-identity is not categorical, studies that have compared storytelling among mothers and fathers have begun to reveal a number of important differences between male and female identified parents. That is, stories learned from fathers may reflect different themes than stories learned from mothers (Fivush, 1989; Fivush et al., 2011). While the most common themes in intergenerational narratives, specifically in adolescence, tend to be relationships, leisure, and medical issues or accidents (Chen et al., 2021), research on gender in narratives about parents provides evidence to show that maternal stories are more elaborative and include more details on emotion, affect, and relationships, whereas paternal ones are more achievement-and self-oriented (Gilligan, 1982; Basow, 1992; Haden et al., 1996; Fivush and Buckner, 2000; Fivush, 2008; Fivush et al., 2011; Grysman et al., 2016). This is not surprising, considering past research demonstrating that females generally express more emotion than males do (Bischoping, 1993; Basow and Rubenfeld, 2003; Leaper and Ayres, 2007; Newman et al., 2008). The gender difference in the content of family stories is reflected in the way that adolescents share family stories; when speaking about mothers (compared to when speaking about fathers) they tell stories that are more elaborative, affiliative, and contain more general affect and specific emotion (Fivush et al., 2011).

As family narrative content tends to be gendered, so does the process by which it is shared. Mothers tend to be more involved in family storytelling than fathers are (Fivush et al., 2011). Additionally, Fivush (1989) found that mothers tend to focus more on positive emotion when talking to daughters, but discuss positive and negative emotions equally with sons. Furthermore, mothers tend to talk more about anger with their sons and more about sadness to their daughters. This may lead to girls feeling that anger is inappropriate for them to express, and similarly, to boys feeling that they should not express sadness. Through the process of intergenerational family narrative sharing, individuals pass down cultural ideals, including gender stereotypes and “gender-appropriate” emotional reactions, ideals that are seamlessly woven into the narrative and as a result, are taught, learned, and maintained through the chain of family narrative transmission across generations (Fivush, 1989; Fivush et al., 2011). Nevertheless, it is important to note that the majority of existing studies on intergenerational narrative sharing were conducted using mostly white, middle-class, heterosexual samples.

### Gendered family stories and wellbeing

The relationship between family narrative sharing and gender may be particularly relevant to mental health across generations. For one, research shows that the difference in the way that girls and boys receive family background knowledge translates to a difference in the way it impacts their wellbeing. Fivush et al. (2011) and Fivush (2011) found that girls, but not boys, who tell maternal intergenerational narratives involving more connections and perspective-taking have higher wellbeing, according to their mothers. Further research on family narrative sharing explores how both content and parent gender may impact the way in which these stories affect wellbeing (Chen et al., 2021). For instance, a study in which parent narratives told by adolescents were coded based on content, theme, and gender of the parent found that participants tended to have higher self-esteem only when they included more subjective perspective in maternal narratives, or when they had a more apparent central theme in paternal narratives. Additionally, adolescents who included a more cohesive theme in narratives about their mothers tended to demonstrate higher depressive symptoms, although it is important to note that this finding was mediated by culture and adolescent age (Chen et al., 2021). The research presented here on gendered differences found in the family narrative may seem to be in line with sexist stereotypes; nevertheless, it is important to keep in mind that intergenerational storytelling is a way of sharing, or passing down, culture, which includes cultural, and potentially stereotypical, ideas surrounding gender.

## Discussion

Past work on the intergenerational transmission of biographical knowledge explores the critical role of the family narrative in regard to one’s wellbeing and one’s identity. In doing so, it sheds light on the variety of factors that impact what kinds of stories we tell, how we tell them, who we tell them to, and ultimately, how knowing about these stories affects our wellbeing. Although still somewhat nascent, there appears to be a convergence of finding indicating that knowledge of one’s family history can play a role in supporting wellbeing and mental health across generations. Such findings can lead to the development of prevention strategies and treatments for mental health concerns as researchers continue to identify the specific factors, practices, and mechanisms underlying these associations. Prior work with children offers considerable promise for the ways in which family stories can be translated into clinical practice. For instance, memory-based interventions have shown that reminiscence style is not fixed. Parents can be coached to share more elaborate and emotionally rich stories with their young children, which in turn can lead to positive cognitive and emotional outcomes among their children (e.g., Valentino et al., 2013; Salmon and Reese, 2016; Van Bergen et al., 2018).

It is still unclear exactly how biographical knowledge affects wellbeing, particularly in relation to cultural practices and factors such as gender, which are distributed through the intergenerational process of family narrative exchange. Components of what is known about this process are scattered between studies that vary in terms of participant age group, mental health measures used, and what is asked about family history, as well as how that knowledge is measured and analyzed. Research on gender and the family narrative is limited, particularly in conjunction with wellbeing and narrative content. Future research should investigate how gender, narrative theme, and narrative content are interrelated and may independently and jointly influence mental health. Does knowledge about mothers have a different impact on wellbeing than knowledge about fathers? Maybe that depends on the gender of the receiver of those stories, the content of the narratives, or how detailed they are in one’s memory. It is also worth conducting this research on young adults, who have an increased sense of self compared to children, and being inclusive of other mental health issues besides depression, such as anxiety, PTSD, and stress, when measuring wellbeing. This is critical in determining exactly how knowing about family may influence wellbeing. For example, knowing more stories about careers and achievement may be linked to increased levels of anxiety, but not depression. Furthermore, a better understanding of how gender and narratives contribute to wellbeing requires greater cultural and contextual diversity. To date, studies on intergenerational narrative sharing have been conducted among mostly white, middle-class, heterosexual samples (however see: Chen et al., 2021; Gardella et al., 2021). Studies are also needed to examine how other expressions of gender identity (e.g., trans and non-binary) may also be linked with intergenerational memory practices and wellbeing.

The existing knowledgebase surrounding family narrative transmission is promising and points to certain areas of significance that require further exploration, particularly by integrating certain factors together and investigating how they may jointly and separately impact wellbeing. Unifying the bits and pieces of what we already know in this area of research is critical to further uncovering how both what we know about our families and who we are is connected to our mental health.

## Data availability statement

The original contributions presented in the study are included in the article, further inquiries can be directed to the corresponding author.

## Author contributions

AE and AB contributed to think conceptualization and writing of the manuscript. All authors contributed to the article and approved the submitted version.

## Funding

This work was financially supported by the Mellon Foundation and the Consortium on Forced Migration, Displacement, and Education.

## Conflict of interest

The authors declare that the research was conducted in the absence of any commercial or financial relationships that could be construed as a potential conflict of interest.

## Publisher’s note

All claims expressed in this article are solely those of the authors and do not necessarily represent those of their affiliated organizations, or those of the publisher, the editors and the reviewers. Any product that may be evaluated in this article, or claim that may be made by its manufacturer, is not guaranteed or endorsed by the publisher.
